# Supplementary material for: Complexome profiling on the Chlamydomonas lpa2 mutant reveals insights into PSII biogenesis and new PSII associated proteins
Source: J Exp Bot. 2021 Aug 26;73(1):245–62. doi: 10.1093/jxb/erab390 (PMC8730698; doi:10.1093/jxb/erab390)
Supplement: erab390_suppl_Supplementary_Dataset_S1 [file erab390_suppl_supplementary_dataset_s1.zip › Supplemental Dataset 1 - Excel List and all profiles/plots/ISU1_Cre13.g568850.html]

Trivial name: ISU1  
  
Euclidean distance: 984.74  
Mean Intensity (WT): 40.90  
Mean Intensity (Mut): 0.00  
Distance: 24.08  
  
MapMan: protein.assembly and cofactor ligation;Co-factor and vitamine metabolism.iron-sulphur clusters  
  
p value of intensity sums Welch test: 0.2017
